# Barriers to Use of Cardio-Kidney Protective Medications in Chronic Kidney Disease: Results of a Nephrology Provider Survey

**DOI:** 10.1016/j.xkme.2026.101474

**Published:** 2026-07-16

**Authors:** Melanie R. Weltman, Linda-Marie U. Lavenburg, Margaret F. Zupa, Manisha Jhamb

**Affiliations:** 1 Department of Pharmacy & Therapeutics, University of Pittsburgh School of Pharmacy, Pittsburgh, PA; 2 Renal-Electrolyte Division, Department of Medicine, University of Pittsburgh, Pittsburgh, PA; 3 Division of Endocrinology and Metabolism, University of Pittsburgh School of Medicine, Pittsburgh, PA; 4 Caring for Complex Chronic Conditions Research Center, University of Pittsburgh, Pittsburgh, PA

To the Editor:

Evidence-based cardio-kidney protective medications, including sodium/glucose cotransporter 2 inhibitors (SGLT2is), glucagon-like peptide 1 receptor agonists (GLP1RAs), and a nonsteroidal mineralocorticoid antagonist (nsMRAs), reduce chronic kidney disease (CKD) progression and cardiovascular morbidity and mortality in individuals with CKD.^1^^,^^2^ However, uptake of these newer cardio-kidney protective medications remains low. Among those with stage 3 CKD and type 2 diabetes, only 40% used SGLT2is, 33% used GLP1RAs, and <2% used nsMRAs from 2019-2024.^3^ Nephrology providers may face unique barriers in providing evidence-based care.^4^ We seek to elucidate barriers experienced by nephrology providers when using evidence-based cardio-kidney protective medications in patients with CKD.

We created an anonymous online survey based on published literature, which was refined with input from 6 stakeholders from nephrology, endocrinology, cardiology, and internal medicine specialties who have experience in caring for patients with CKD (Item S1).^5^, ^6^, ^7^ The survey was designed to take 5-10 minutes to complete. It collected demographic and practice setting information and assessed perceived knowledge and beliefs about SGLT2is, GLP1RAs, and nsMRAs using a Likert scale. Providers were asked “Which of the following factors are key barriers for you while using cardio-kidney protective medications in people with CKD?” for each medication class across provider-level, medication-level, patient-level, and logistical factor categories. An “Other” response allowed for free-text input (see online supplement). The survey was distributed to US nephrology providers (nephrologists and advanced practice providers) between April and June 2025, through professional networks, word of mouth, and electronic mailing lists, including distribution via the National Kidney Foundation. Responses were analyzed descriptively using frequencies and percentages. Free-text responses were coded to corresponding answer options, when appropriate, or included as other. This study was approved by the institutional review board of the University of Pittsburgh and determined exempt research under 45 CFR 46.104(d).

We received 61 responses from 44 nephrologists and 17 advanced practice providers. Forty-six percent of participants had >15 years of experience (Table S1). The most common practice sites were academic (25%) and nonacademic group practices (58%). Nearly half (47%) prescribed SGLT2is >5 times monthly, but only 10% and 5% prescribed GLP1RAs and nsMRAs >5 times monthly; 52% and 39% reported never prescribing GLP1RAs and nsMRAs (Figs S1 and S2). Most endorsed knowledge of drug mechanisms, side effects, and indications (Fig 1). Although most were comfortable prescribing SGLT2is and nsMRAs (93% and 77%, respectively), only about half were comfortable prescribing GLP1RAs. Common barriers to SGLT2i use were cost (74%), polypharmacy (66%), and patient preferences/hesitation (66%); GLP1RA use were cost (84%), prior authorization (70%), and uncertainty about prescribing responsibility (70%); and nsMRA use were polypharmacy (66%) and cost (57%; Fig 2). Descriptive results by subgroups of provider type and experience are presented in Figs S3-S7. In general, compared with physicians, advanced practice providers reported less comfort with prescribing and less knowledge about medications of interest, especially nsMRAs.

**Figure 1:**
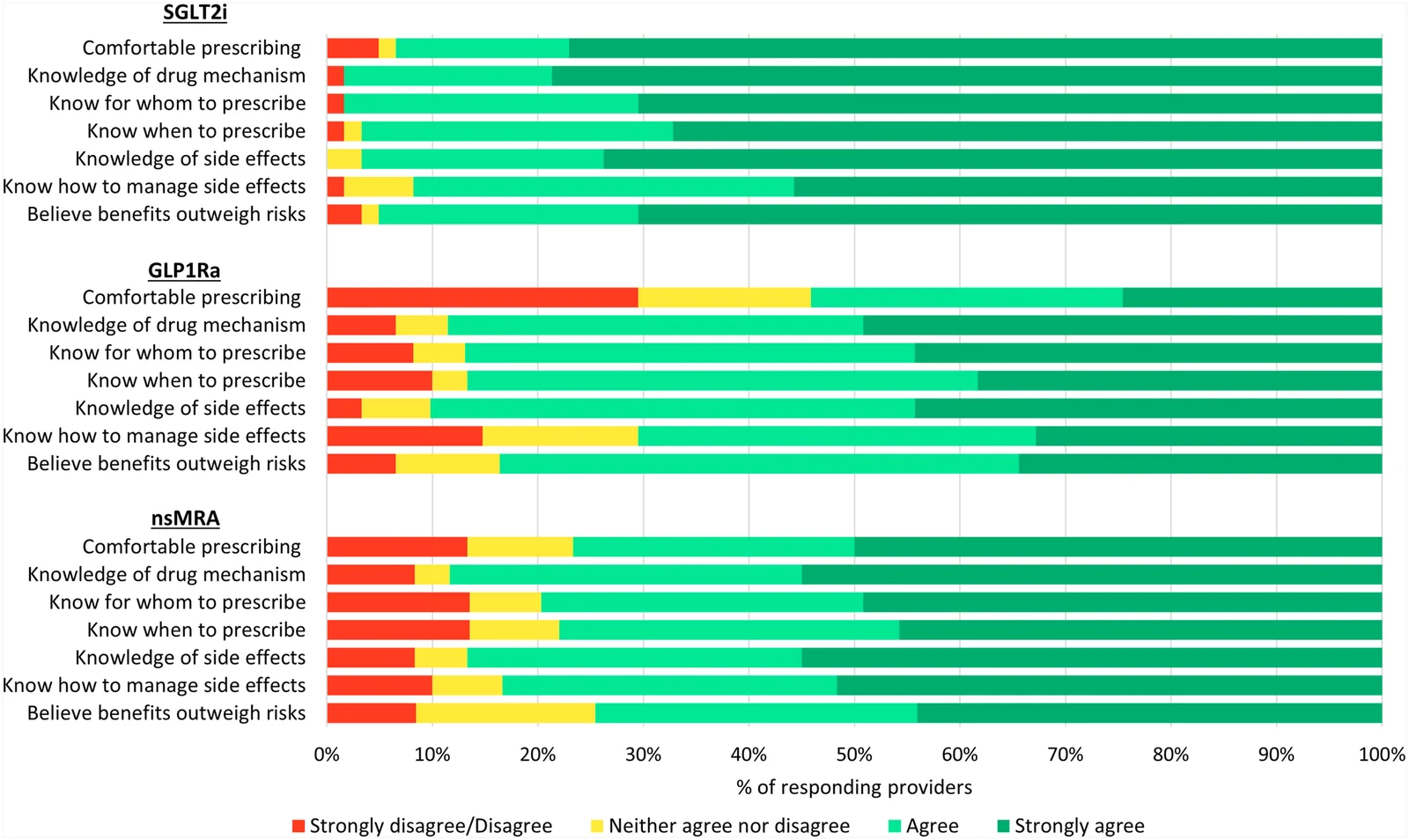
Perceived knowledge and beliefs about cardio-kidney protective medications. Data from survey items 7, 13, and 19 (see online supplement). GLP1RA, glucagon-like peptide-1 receptor agonist; nsMRA, nonsteroidal mineralocorticoid antagonist; SGLT2i, sodium/glucose cotransporter 2 inhibitor.

**Figure 2:**
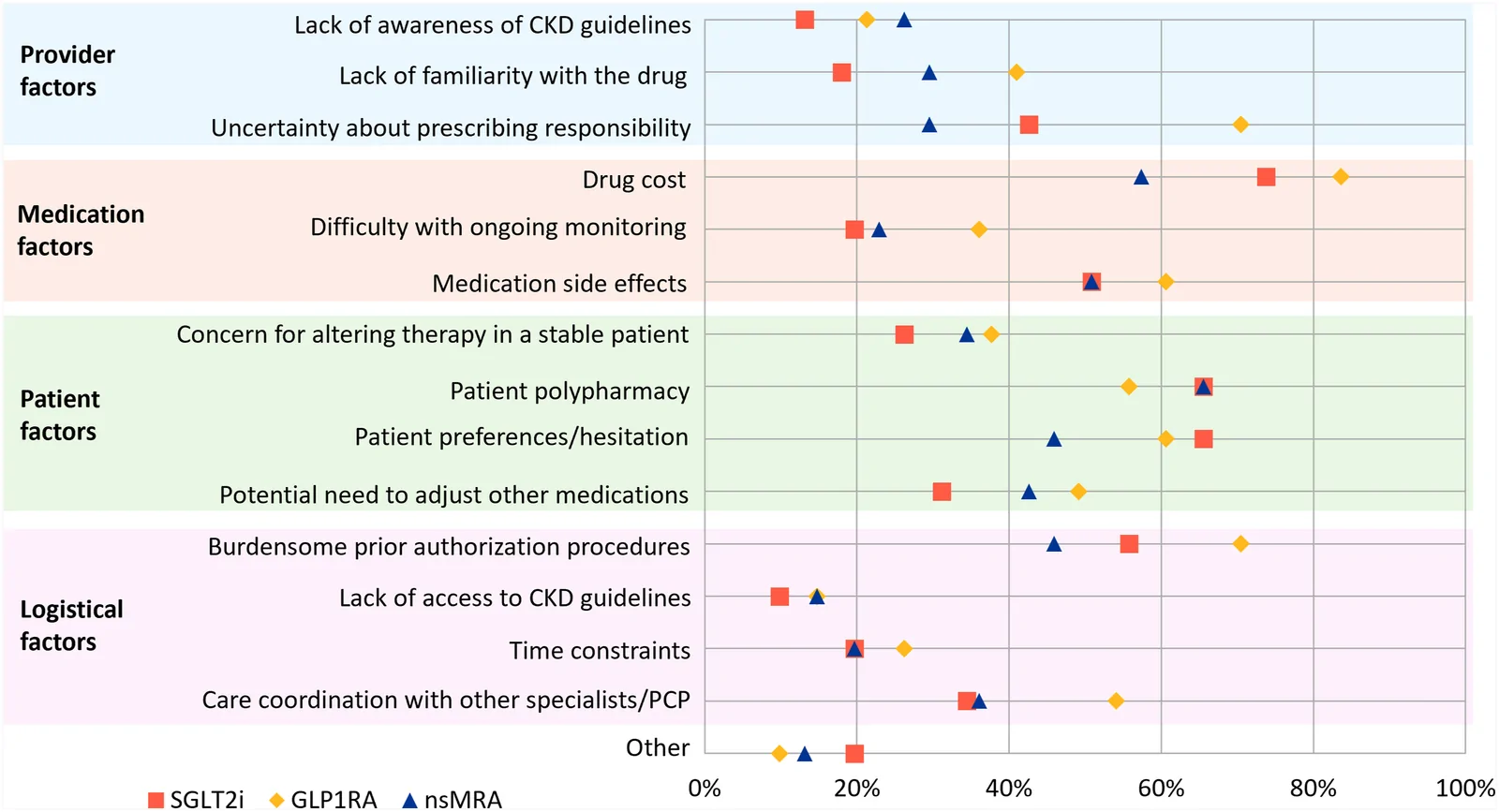
Barriers to using cardio-kidney protective medications. Data from survey items 8-11, 14-17, and 20-23 (see online supplement). CKD, chronic kidney disease; GLP1RA, glucagon-like peptide-1 receptor agonist; nsMRA, nonsteroidal mineralocorticoid antagonist; PCP, primary care provider; SGLT2i, sodium/glucose cotransporter 2 inhibitor.

Cost was one of the most reported barriers across all medication classes. Manufacturer-sponsored savings programs may help with affordability, but programs are limited by restrictive qualifying criteria (eg, insurance type, income limits), lack of provider awareness, and insufficient time or system-based resources, such as a pharmacist or social worker, to help patients apply. However, given the effectiveness of these medications in improving high-cost outcomes like hospitalizations, cardiovascular interventions, and dialysis, there is an urgent need for innovative value-based and alternative payment models of care to improve equitable medication access and affordability.

Multidisciplinary team-based care can address many of the additional barriers reported. For example, pharmacists can address polypharmacy through comprehensive medication management, identify medication assistance programs, help with dose titration, facilitate care coordination, address patient hesitancy, and lead educational and experiential learning opportunities to support nephrology provider confidence and prescribing self-efficacy. Integration of pharmacists into the kidney care team has been supported by major organizations including Kidney Disease: Improving Global Outcomes (KDIGO) and the National Kidney Foundation, as well as by leaders in clinical pharmacy.^8^ Support from other team members, such as social workers (to address access to health care cost resources) and medical assistants, nurses, and other clinic staff (for prior authorization procedures, patient education, and care coordination) is likely needed to change prescribing practices. Lastly, continued efforts from national organizations, health systems, and payors are needed to advance education and promote ownership and prescribing of evidence-based cardio-kidney protective medications in nephrology, much like what has been called for in the cardiology community.^9^

Our study is limited by potential self-selection bias and inability to accurately calculate response rate. Additionally, the small number of responses, largely from academic or group practice clinicians, and sampling of foundation members and volunteers may limit the generalizability of our results. In future work, we plan to further explore barriers to care through in-depth qualitative interviews with patients, providers, and payors.

Nephrology providers experience multilevel barriers to using evidence-based cardio-kidney protective medications in patients with CKD. This study can inform future interventions targeting CKD medication optimization.

## Supplementary Material

Supplementary File (PDF) Table S1; Figure S1-S7; Item S1.
