# Supplementary material for: Barriers to Use of Cardio-Kidney Protective Medications in Chronic Kidney Disease: Results of a Nephrology Provider Survey
Source: Kidney Med. 2026 Jul 16;8(10):101474. doi: 10.1016/j.xkme.2026.101474 (PMC13635260; doi:10.1016/j.xkme.2026.101474)
Supplement: Supplementary file 1 [file mmc1.pdf]

Supplementary appendix

Barriers to use of cardio-kidney protective medications in chronic kidney disease: Results of a nephrology provider survey

Melanie R. Weltman, Linda-Marie U. Lavenburg, Margaret F. Zupa, Manisha Jhamb

Contents

Table S1. Characteristics of participants 2

Figure S1. Prescribing frequency of cardio-kidney protective medications 3

Figure S2. Prescribing frequency of cardio-kidney protective medications by patient volume 4

Figure S3. Prescribing frequency of cardio-kidney protective medications by years of experience 5

Figure S4. Perceived knowledge and beliefs about cardio-kidney protective medications by provider type 6

Figure S5. Barriers to using cardio-kidney protective medications by provider type 7

Figure S6. Perceived knowledge and beliefs about cardio-kidney protective medications by provider years in practice 8

Figure S7. Barriers to using cardio-kidney protective medications by provider years in practice 9

Item S1. Survey of nephrology providers: Barriers to use of cardio-kidney protective medications in chronic kidney disease (CKD) 10

Table S1. Characteristics of participants

**Characteristics**	***n* (%)**
Provider type	
Nephrologist	44 (72)
APP (PA or NP)	17 (28)
Years of service	
0-5	17 (28)
6-10	11 (18)
11-15	5 (8)
>15	28 (46)
Setting	
Academic	15 (25)
Government	2 (3)
Hospital-based	3 (5)
Multispecialty group	15 (25)
Single specialty group	20 (33)
Solo private practice	1 (2)
Other[a]	5 (8)
Number of providers in practice	
2-5	14 (23)
6-10	18 (30)
11-50	24 (39)
>50	5 (8)
CKD patients per week	
20 or less	22 (36)
21-40	24 (39)
41-60	6 (10)
>60	9 (15)

[a]Other setting included inpatient, managed care, unspecified specialty practice, transplant

Figure S1. Prescribing frequency of cardio-kidney protective medications

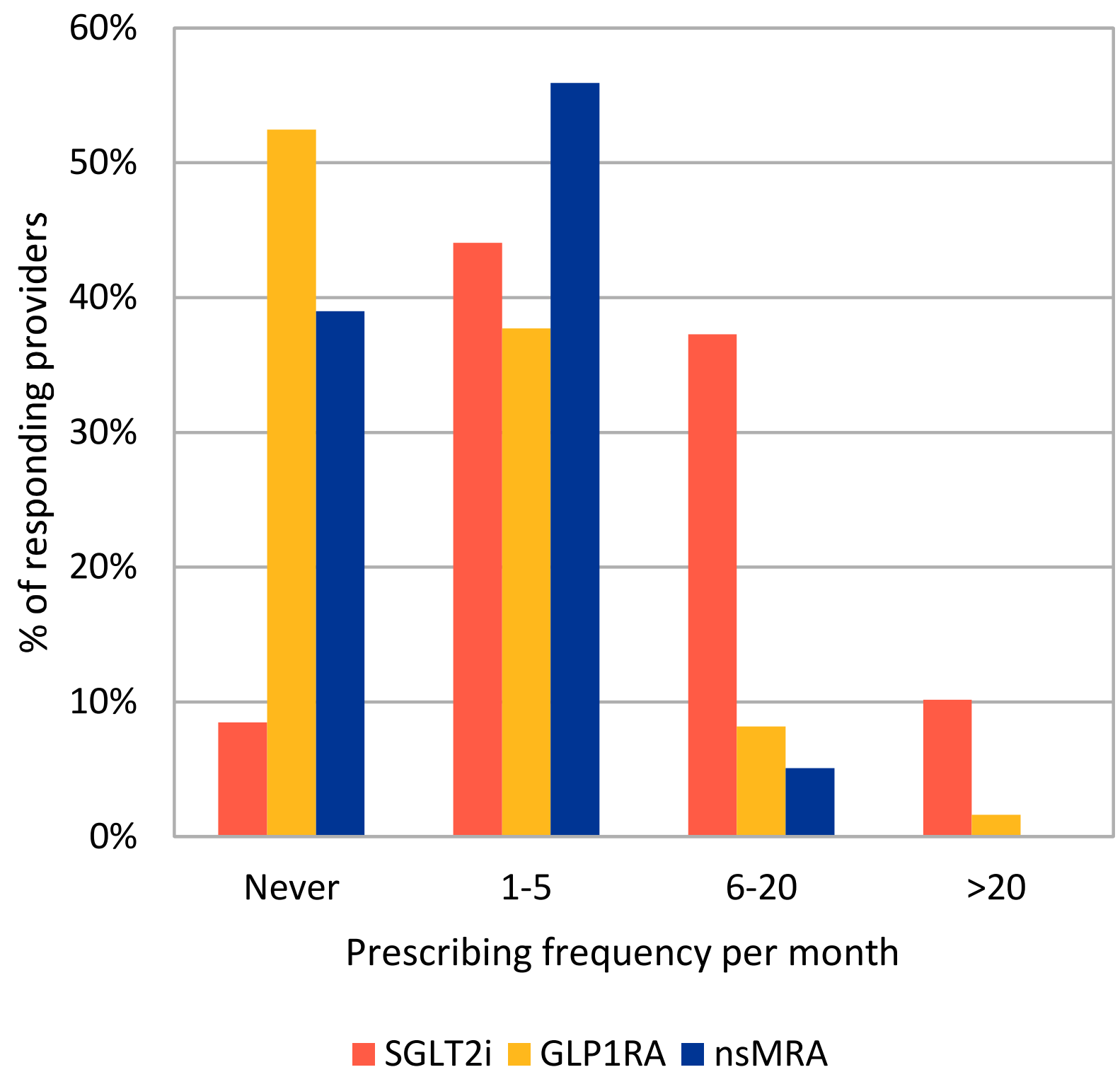

Figure S2. Prescribing frequency of cardio-kidney protective medications by patient volume

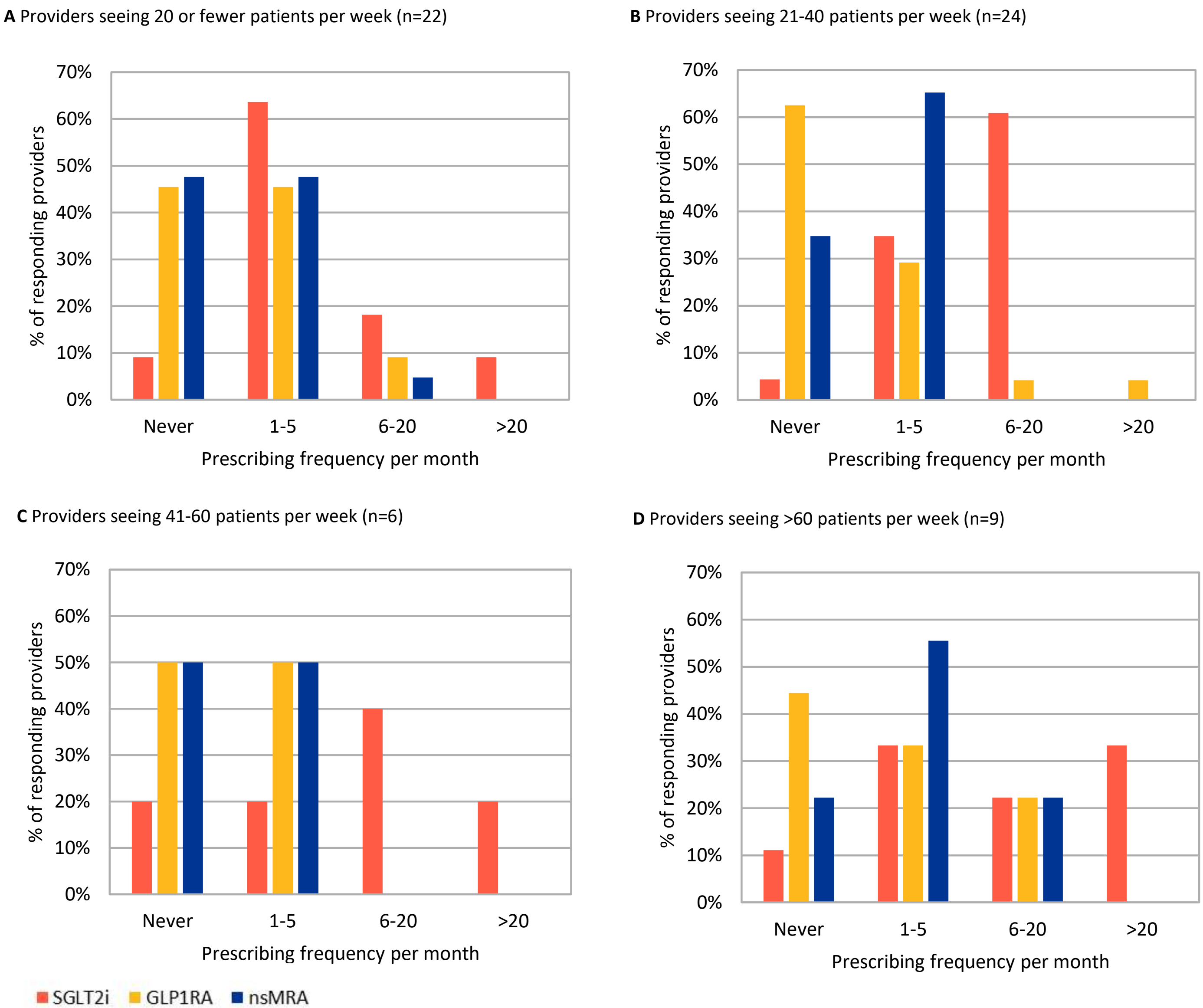

Figure S3. Prescribing frequency of cardio-kidney protective medications by years of experience

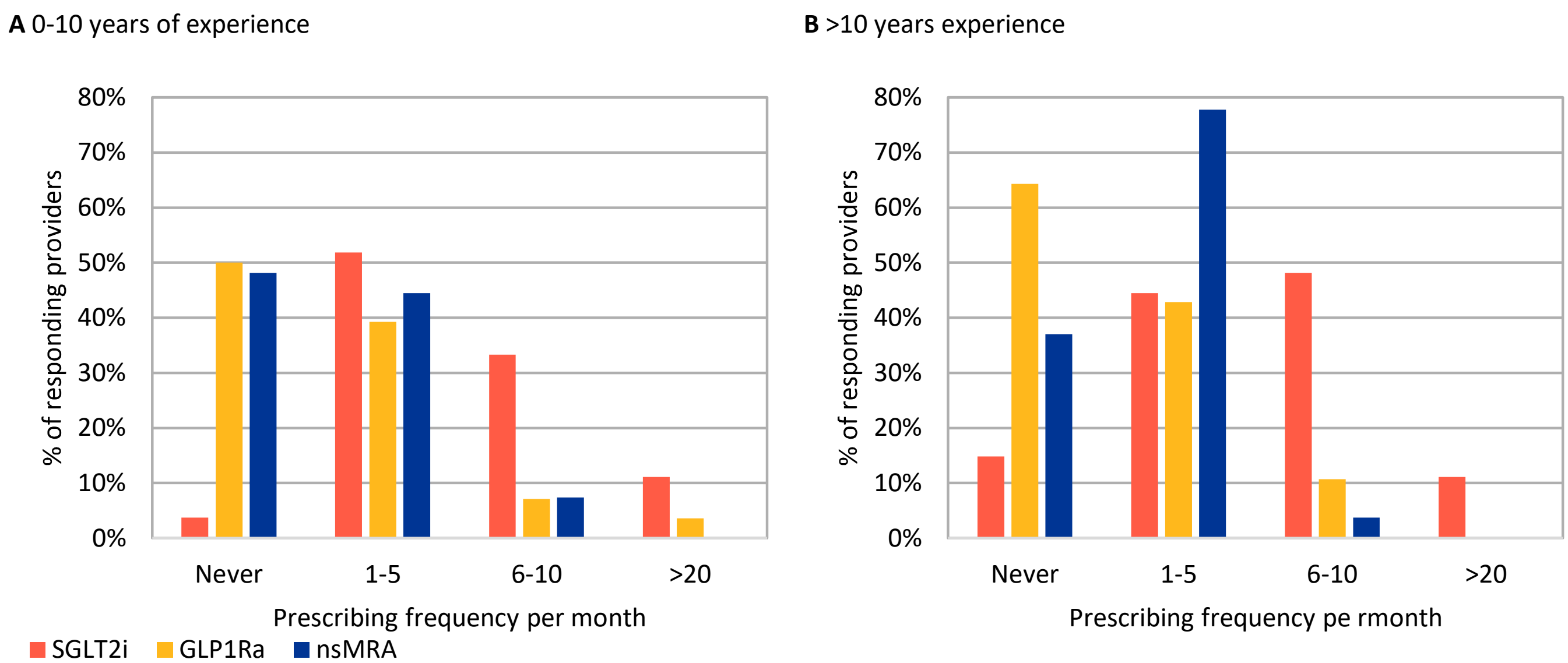

Figure S4. Perceived knowledge and beliefs about cardio-kidney protective medications by provider type

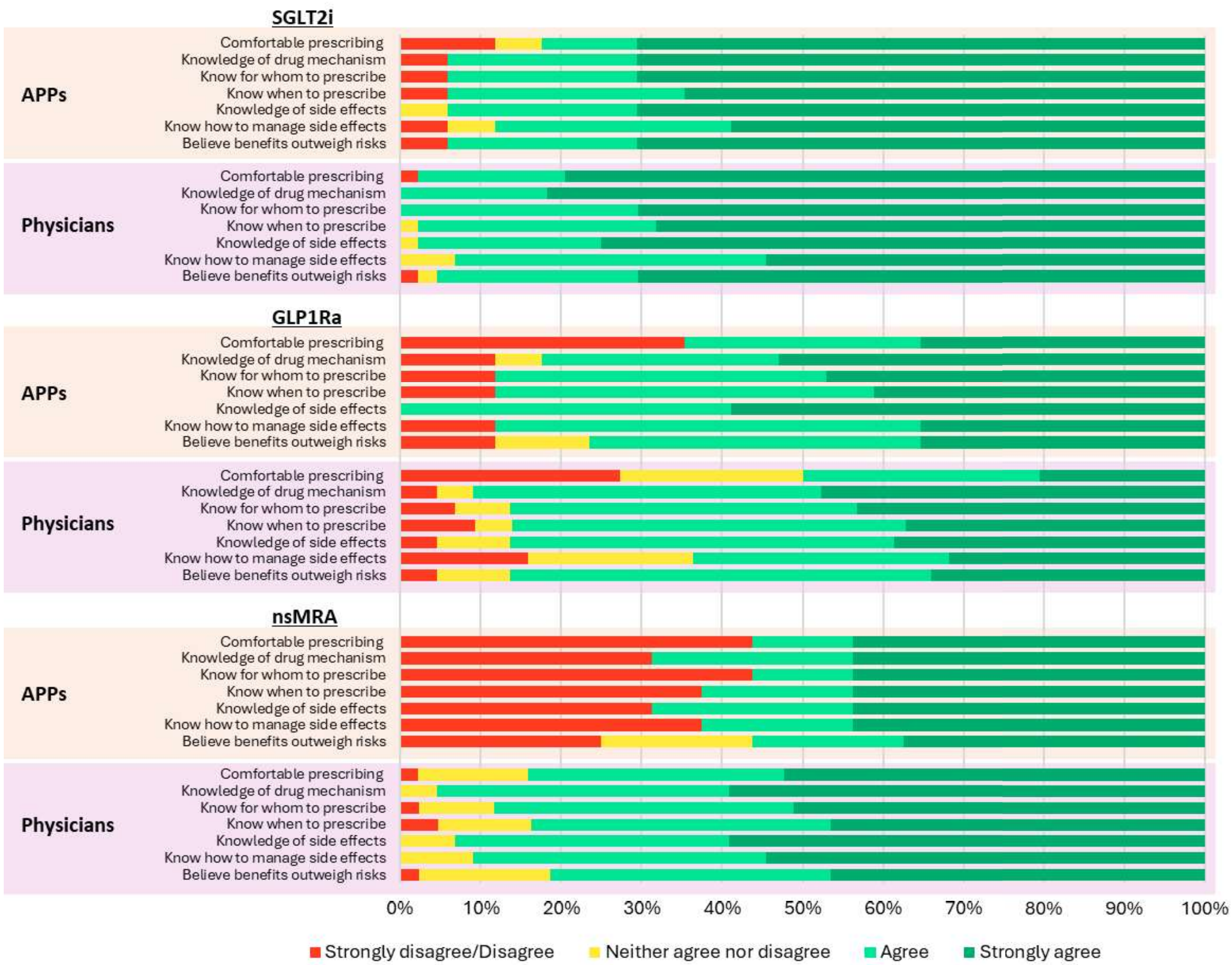

Physicians, n=44; APPs (advanced practice providers), n=17

Figure S5. Barriers to using cardio-kidney protective medications by provider type

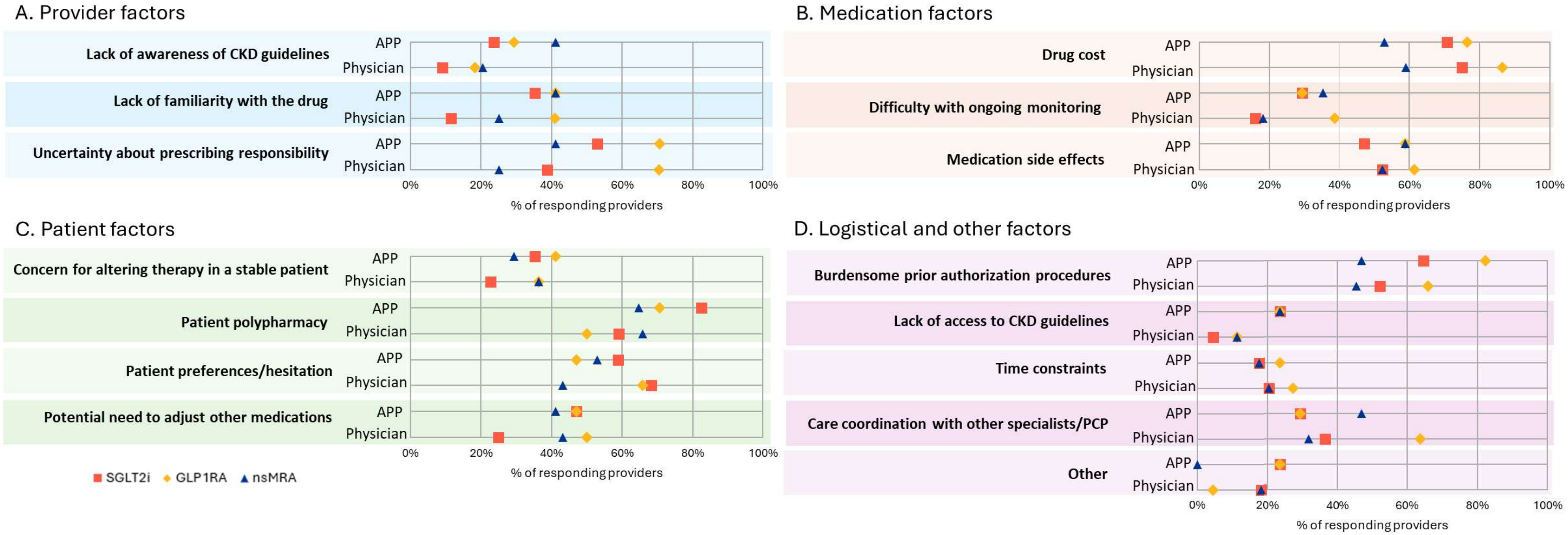

Physicians, n=44; APPs (advanced practice providers), n=17

Figure S6. Perceived knowledge and beliefs about cardio-kidney protective medications by provider years in practice

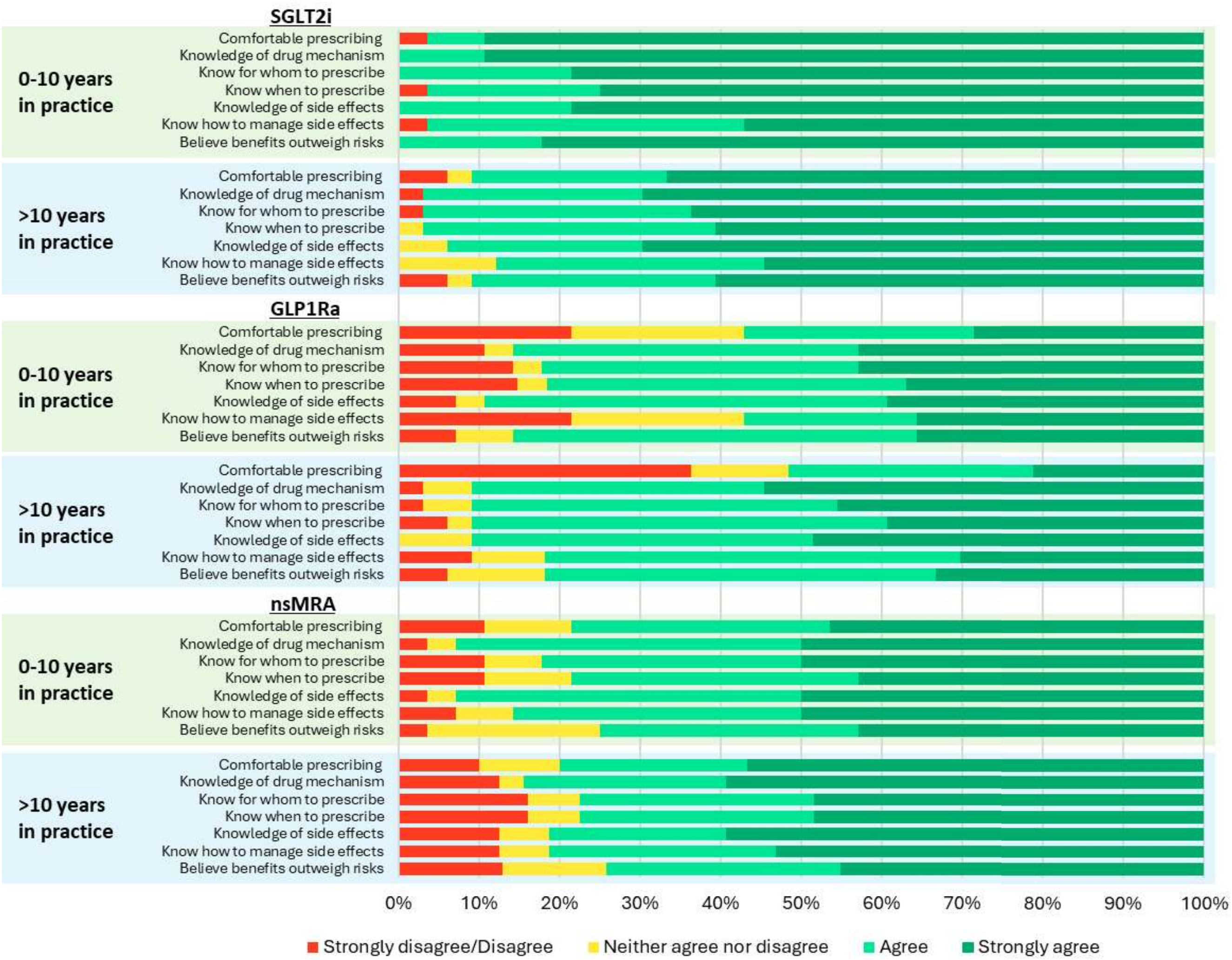

0-10 years, n=28; >10 years, n=33

Figure S7. Barriers to using cardio-kidney protective medications by provider years in practice

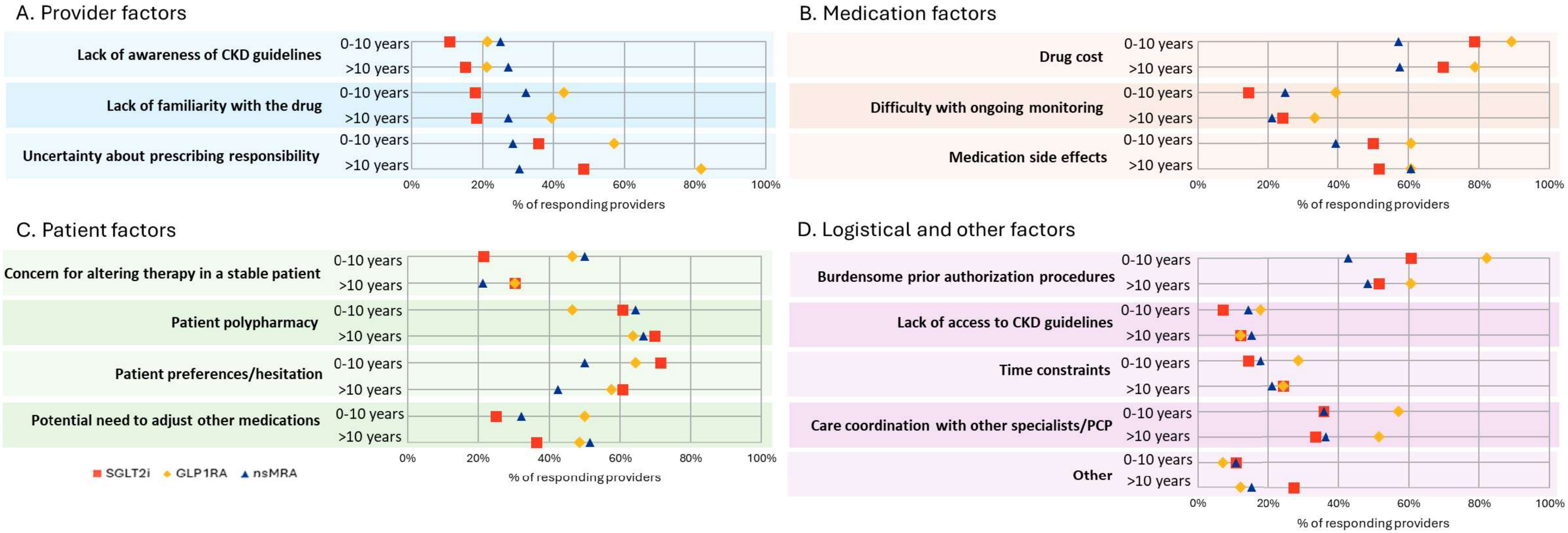

0-10 years, n=28; >10 years, n=33

Item S1. Survey of nephrology providers: Barriers to use of cardio-kidney protective medications in chronic kidney disease (CKD)

The purpose of this research study is to elucidate barriers to prescribing of newer cardio-kidney protective medications (i.e., SGLT-2 inhibitors, GLP-1 receptor agonists, and non-steroidal mineralocorticoid receptor antagonists (nsMRA)) by nephrology clinic providers. Use of newer cardio-kidney protective medications in people with chronic kidney disease (CKD) is suboptimal. Barriers to prescribing these medications for people with CKD in primary care and other specialty settings have been described in literature, but knowledge of barriers and facilitators specific to nephrology clinics limited.

We will be surveying 200 nephrology providers (nephrologists and advanced practice providers) who practice in the United States using an internet-based questionnaire. The questionnaire will focus on questions regarding current prescribing practices, comfort level with newer cardio-kidney protective medications, and barriers to prescribing these medications. The questionnaire will also gather basic information regarding provider training, years of service, and practice setting. No identifiable information will be gathered. The questionnaire will take approximately 5-10 minutes to complete.

There are no foreseeable risks, nor are there direct benefits to participants completing the questionnaire. Participation is voluntary, and respondents will not receive any incentive for participation. Participants can withdraw from the survey at any time by closing the web browser. If they choose not to participate or complete the questionnaire, their relationship with the study team will not be affected. Anonymous survey data may be shared for future research without additional consent.

1. What best describes your professional training?
    a. Advanced practice provider (PA or NP)
    b. Nephrologist
    c. Nephrology fellow
    d. Other (please specify)
2. How many years have you been in practice?
    a. 0-5
    b. 6-10
    c. 11-15
    d. More than 15
3. Which of the following best describes your outpatient practice setting?
    a. Solo private practice
    b. Single specialty group practice
    c. Multispecialty group practice
    d. Other (please specify)

4. Including you, how many providers are in your practice?
    a. 1
    b. 2-5
    c. 6-10
    d. 11-50
    e. More than 50
5. How many patients with chronic kidney disease (CKD) do you see **per week** in your outpatient CKD clinic?
    a. 20 or less
    b. 21-40
    c. 41-60
    d. More than 60

Now, think about how you use SGLT-2 inhibitors (e.g., empagliflozin, dapagliflozin) in your current practice.

6. How often do you personally prescribe SGLT-2 inhibitors for CKD?
    a. Never
    b. 1-5 times per month
    c. 6-20 times per month
    d. More than 20 times per month
7. Please rate your agreement with the following statements about SGLT-2 inhibitors in CKD:

	Strongly Agree	Agree	Neither Agree nor Disagree	Disagree	Strongly Disagree
I am comfortable prescribing SGLT-2 inhibitors for patients with CKD					
I know what SGLT-2 inhibitors do and how they work					
I know for which patients current evidence supports SGLT-2 inhibitor benefit and safety					
I know when in the course of a patient's care SGLT-2 inhibitor therapy should be initiated					
I know what the potential side effects of SGLT-2 inhibitors are					
I know how to manage the potential side effects of SGLT-2 inhibitors					
I believe that SGLT-2 inhibitor benefits outweigh the risks for most CKD patients					

8. Which of the following **provider factors** are key barriers for you while using SGLT-2 inhibitors in people with CKD? (choose all that apply)
    ☐ Lack of awareness of CKD guidelines (e.g., KDIGO)
    ☐ Lack of familiarity with the drug
    ☐ Uncertainty about whose responsibility it is to prescribe (e.g., when a patient is seeing primary care and other specialists)
    ☐ Others (please specify)
    ☐ None of the above

9. Which of the following **medication factors** are key barriers for you while using SGLT-2 inhibitors in people with CKD? (choose all that apply)
    ☐ Cost of medication
    ☐ Difficulties with ongoing monitoring
    ☐ Medication adverse effects
    ☐ Others (please specify)
    ☐ None of the above

10. Which of the following **patient factors** are key barriers for you while using SGLT-2 inhibitors in people with CKD? (choose all that apply)
    ☐ Concern for altering management plan in a stable patient
    ☐ Patient polypharmacy
    ☐ Patient preferences/hesitation
    ☐ Potential need to adjust other medications (e.g. concurrent diabetes medications)
    ☐ Others (please specify)
    ☐ None of the above

11. Which of the following **logistical factors** are key barriers for you while using SGLT-2 inhibitors in people with CKD? (choose all that apply)
    ☐ Burdensome prior authorization procedures
    ☐ Lack of access to CKD guidelines (e.g., KDIGO)
    ☐ Time constraints
    ☐ Too hard to coordinate plan of care with other specialists and primary care provider
    ☐ Others (please specify)
    ☐ None of the above

Now, think about how you use GLP-1 receptor agonists (e.g., semaglutide, dulaglutide, tirzepatide) in your current practice.

12. How often do you personally prescribe GLP-1 receptor agonists for patients with CKD?
    a. Never
    b. 1-5 times per month
    c. 6-20 times per month
    d. More than 20 times per month

13. Please rate your agreement with the following statements about GLP-1 receptor agonists in CKD:

	Strongly Agree	Agree	Neither Agree nor Disagree	Disagree	Strongly Disagree
I am comfortable prescribing GLP-1 receptor agonists for patients with CKD					
I know what GLP-1 receptor agonists do and how they work					
I know for which patients current evidence supports GLP-1 receptor agonist benefit and safety					
I know when in the course of a patient's care GLP-1 receptor agonist therapy should be initiated					
I know what the potential side effects of GLP-1 receptor agonists are					
I know how to manage the potential side effects of GLP-1 receptor agonists					
I believe that GLP-1 receptor agonist benefits outweigh the risks for most CKD patients					

14. Which of the following **provider factors** are key barriers for you while using GLP-1 receptor agonists in people with CKD? (choose all that apply)
    - ☐ Lack of awareness of CKD guidelines (e.g., KDIGO)
    - ☐ Lack of familiarity with the drug
    - ☐ Uncertainty about whose responsibility it is to prescribe (e.g., when a patient is seeing primary care and other specialists)
    - ☐ Others (please specify)
    - ☐ None of the above

15. Which of the following **medication factors** are key barriers for you while using GLP-1 receptor agonists in people with CKD? (choose all that apply)
    - ☐ Cost of medication
    - ☐ Difficulties with ongoing monitoring
    - ☐ Medication adverse effects
    - ☐ Others (please specify)
    - ☐ None of the above

16. Which of the following **patient factors** are key barriers for you while using GLP-1 receptor agonists in people with CKD? (choose all that apply)
    - ☐ Concern for altering management plan in a stable patient
    - ☐ Patient polypharmacy
    - ☐ Patient preferences/hesitation
    - ☐ Potential need to adjust other medications (e.g. concurrent diabetes medications)
    - ☐ Others (please specify)
    - ☐ None of the above
17. Which of the following **logistical factors** are key barriers for you while using GLP-1 receptor agonists in people with CKD? (choose all that apply)
    - ☐ Burdensome prior authorization procedures
    - ☐ Lack of access to CKD guidelines (e.g., KDIGO)
    - ☐ Time constraints
    - ☐ Too hard to coordinate plan of care with other specialists and primary care provider
    - ☐ Others (please specify)
    - ☐ None of the above

Now, think about how you use non-steroidal mineralocorticoid receptor antagonists (ns-MRA, e.g., finerenone) in your current practice.

18. How often do you personally prescribe ns-MRA for patients with CKD?
    a. Never
    b. 1-5 times per month
    c. 6-20 times per month
    d. More than 20 times per month
19. Please rate your agreement with the following statements about ns-MRA in CKD:

	Strongly Agree	Agree	Neither Agree nor Disagree	Disagree	Strongly Disagree
I am comfortable prescribing ns-MRA for patients with CKD					
I know what ns-MRA do and how they work					
I know for which patients current evidence supports ns-MRA benefit and safety					
I know when in the course of a patient's care ns-MRA therapy should be initiated					
I know what the potential side effects of ns-MRA are					
I know how to manage the potential side effects of ns-MRA					
I believe that ns-MRA benefits outweigh the risks for most CKD patients					

20. Which of the following **provider factors** are key barriers for you while using ns-MRA in people with CKD? (choose all that apply)
    - ☐ Lack of awareness of CKD guidelines (e.g., KDIGO)
    - ☐ Lack of familiarity with the drug
    - ☐ Uncertainty about whose responsibility it is to prescribe (e.g., when a patient is seeing primary care and other specialists)
    - ☐ Others (please specify)
    - ☐ None of the above
21. Which of the following **medication factors** are key barriers for you while using ns-MRA in people with CKD? (choose all that apply)
    - ☐ Cost of medication
    - ☐ Difficulties with ongoing monitoring
    - ☐ Medication adverse effects
    - ☐ Others (please specify)
    - ☐ None of the above
22. Which of the following **patient factors** are key barriers for you while using ns-MRA in people with CKD? (choose all that apply)
    - ☐ Concern for altering management plan in a stable patient
    - ☐ Patient polypharmacy
    - ☐ Patient preferences/hesitation
    - ☐ Potential need to adjust other medications (e.g. concurrent diabetes medications)
    - ☐ Others (please specify)
    - ☐ None of the above
23. Which of the following **logistical factors** are key barriers for you while using ns-MRA in people with CKD? (choose all that apply)
    - ☐ Burdensome prior authorization procedures
    - ☐ Lack of access to CKD guidelines (e.g., KDIGO)
    - ☐ Time constraints
    - ☐ Too hard to coordinate plan of care with other specialists and primary care provider
    - ☐ Others (please specify)
    - ☐ None of the above
